# Development and psychometric evaluation of an emotional growth questionnaire for university students

**DOI:** 10.15171/hpp.2018.43

**Published:** 2018-10-27

**Authors:** Fatemeh Rostami, Jamileh Mokhtari Nouri, Abbas Ebadi, Morteza Khaghanizade

**Affiliations:** ^1^Behavioral Sciences Research Center (BSRC), Faculty of Nursing, Baqiyatallah University of Medical Sciences, Tehran, Iran; ^2^Behavioral Sciences Research Center, Life style institute, Faculty of Nursing, Baqiyatallah University of Medical Sciences, Tehran, IR Iran; ^3^Behavioral Sciences Research Center, Baqiyatallah University of Medical Sciences, Tehran, Iran

**Keywords:** Emotional growth, Reliability, Validity, Psychometric, Confirmatory factor analysis, Exploratory factor analysis

## Abstract

**Background:** Despite the importance of emotional growth promotion among students, there is still a lack of standard emotional growth tools to evaluate the concept while developing interventional programs. The aim of present study was to devise and provide a valid and reliable instrument for measuring students’ emotional growth.

**Methods:** This study was conducted from 2016 to 2017 among university students. A questionnaire containing 79 items was made using focus group discussions with students and studying relevant texts. Exploratory factor analysis (EFA) (n = 188) was used to determine the construct validity of the questionnaire. Confirmatory factor analysis (CFA) (n = 38) was performed to assess the validity of the model. Reliability (n = 56) and repeatability (n = 31) of the questionnaire were assessed.

**Results:** A 4-factor (positive identity, self-esteem, effective communication and motivation)and 21-item solution was found as the best solution, which explained 63.5% of total variancebetween the items. The results of CFA approved the validity of the model (CFI = 0.914, RMSEA= 0.070). Cronbach alpha (0.93) and Intra class correlation coefficient (0.91) approved the reliability and repeatability of questionnaire.

**Conclusion:** The 21 items questionnaire seems to be a valid and reliable instrument to measure emotional growth among university students.

## Introduction

Higher education is generally important for developing the scientific knowledge of the students. The university administrators, however, are frequently faced with the criticism that they do not prepare the students for taking various kinds of roles like leadership, which is the essential requirement of organizations.^1^ This means that the university graduates do not acquire emotional and social skills along with the scientific knowledge of their field of study. Universities should take into account the all-inclusive growth of the students embracing all aspects (mental, emotional, and social) of their lives in order to better prepare the graduate students for achieving success in the future. This urges the instructors who approach learning as a process, to participate the students in both teaching and learning processes, and to link an effective association between the students and instructors.^2,3^ Those students who can develop their capabilities to perceive themselves and their surrounding world may create significant relationships and positive changes in their university, work, and life.^4^

According to education theorists, education should approve latent learning, which occurs simultaneously with the routine educational programs. It should also be able to promote and facilitate the development of various human aspects of the students.^5^ For example, in modern nursing, nurses are expected to consider comprehensive nursing care in accordance with the biological, mental, and spiritual needs of patients.^6,7^ However, official nursing education programs in Iran follow a biomedical approach that is mainly focused on medical problems.^8,9^ These official education programs put their focus on conveying the knowledge and mastery of patients’ physical care-related techniques. Besides official programs, students also learn through unofficial latent nursing education curricula. Direct observation of situations such as birth, death, loss, and pain has a significant effect on emotional growth (also referred to as emotional development) and learning among students.^10^ In addition, through interacting with each other as well as with instructors and nursing staff, students acquire some experiences that are associated with long-term effects during their lifetime.^11^

Self-flourishing is the objective of many human-oriented programs. The main emphasis of such programs is on the fact that learning experiences should provide the students with cognitive and emotional learning processes which are inclined with their current lives. Thus, learning these concepts has been integrated in emotional growth.^12^ In emotional growth, the individuals are expected to achieve the ability to control, receive, and recognize feelings and, then, behave accordingly. An emotionally full-grown individual features the capability to properly communicate with others in his/her social life as well as the capability to accept the responsibilities of both himself/herself and others. Moreover, such an individual is successful in interacting with others, is not selfish and self-oriented, and also has the ability to get adapted to the surrounding environment and conditions.^13^ Emotional growth means to have a sense of sympathy, confidence, and competency in making relationships with others and accepting roles of others.^14^ Furthermore, emotional growth is achievable through “effective relationships”, “inducing sense of positive identity”, and “loving others”, which represent “effective interactions”.^15^

Considering the importance of promoting students’ emotional growth as well as the necessity of assessing interventional programs on the students’ emotional growth, it seems definitely essential to have a valid and reliable tool. However, to our knowledge, there is no standard tool for evaluating the emotional growth of university students. Therefore, the present study is aimed to devise and provide a standard, valid, and reliable instrument for measuring students’ emotional growth, so that it can be applicable for improving quality of education among university students.

## Materials and Methods

This mixed-method study was conducted from 2016 to 2017. The participants in the study were undergraduate students from the faculties of nursing in Tabriz University of Medical Sciences and Baqiyatallh University of Medical Sciences‏.

### Questionnaire development

To prepare the questionnaire, the codes obtained from two performed focus group discussions with 11 students (4 female and 7 males) by using conventional qualitative content analysis were extracted. Applying these codes and the items found after comprehensive review of the literature (including the Hermans achievement motivation questionnaire^16^ included 29 questions, Eysenck self-esteem inventory,^17^ 30 questions, Quondam communication skills test^18^ including 34 questions and Oxford Happiness Questionnaire,^19^ 29 questions), a pool of questions containing 79 items was made‏.

### Content validity

At first, a questionnaire with 36 items was prepared by the research team from the pool of questions. Then, to determine its validity, qualitative and quantitative content validity assessment were used. So, the designed questionnaire attached with an answer sheet for the opinions on relevancy and clarity of the questionnaire were sent to the following experts: 7 experts in the field of nursing education (content expert), 3 experts on the questionnaire designing and methodology (methodology expert), 3 lecturer and mentor experts in the field of nursing education and 8 alert students (as lay experts). For the purpose of assessing the relevancy of the questionnaire, individuals were asked to answer the following question: “How much this question is associated with the measured parameter? In other words, how appropriate is this question?” The proposed answers were ranked as follows: 1- not suitable, 2- moderately suitable, 3- suitable and 4- completely suitable. For examining the clarity of the questionnaire, they were asked to answer the following question: “How clear is the meaning of this question?” The proposed answers were categorized as follows: 1- ambiguous, 2- moderately ambiguous, 3- clear and 4- completely clear. Answers 3 and 4 were considered as favorable and answers 1 and 2 were considered as unfavorable, then the relevancy and clarity percentages for two groups (experts and lay experts) were separately measured.

According to the results of this phase, 8 questions were deleted and a 28- items questionnaire was developed. The answer choices for the questionnaire was based on a 6-point scaling: “completely agree” (6), “agree” (5), “somewhat agree” (4), “somewhat disagree” (3), “disagree” (2) and “completely disagree” (1). Three examples of the questions were as follow: 1- I feel particularly pleased with the way I am, 2- My life is purposeful, and 3- I have a good effect on others.

### Construct validity

Exploratory factor analysis (EFA) was used to determine the constructs of the questionnaire. Kaiser-Meyer-Olkin (KMO) and Bartlett’s test of Sphericity tests were used to verify the sampling adequacy and to determine construct validity and appropriateness of data, respectively. Principle component analysis (extraction method) with Varimax rotation and cut-off values ≥0.5 was considered in this analysis. The sample size for this analysis was 188 students (115 females and 75 males). This analysis was performed in SPSS-22 (IBM Corp. Released 2013) software.

Confirmatory factor analysis (CFA) was performed using Stata 14 (StataCorp., College Station, TX, USA) to assess the validity of the EFA model. The sample size for this analysis was 238 students (140 females and 98 males).

### Reliability 

The reliability and repeatability of the questionnaire was assessed using Cronbach’s alpha as measure of internal consistency in a sample of 56 students and intra-class correlation coefficient (ICC) as a measure of repeatability in a sample of 31 students within a two-week interval.

## Results

In terms of content validity, the percentage of item relevancy for the scale was 100%. The level of clarity among the experts and lay experts were 85% and 91%, respectively.

The researchers had no prior idea about which or how many underlying factors could be found to explain the data. Therefore, EFA was considered to be conducted. In the correlation matrix, 28 items were inter-correlated with Pearson coefficient scores of r = 0.132-0.764. KMO measure of sampling adequacy of 0.912 and Bartlett’s test of Sphericity (χ^2^ value 2658.6, *df* = 378, *P* < 0.001) indicated that the EFA was possible. Table 1 shows the items, their mean and standard deviation, and factor loading of four-factor structure of the student emotional growth questionnaire. This 21-item structure was found to explain 63.5% of the variance (See Figure S1, Supplementary materials).

The items were extracted in four factors: positive identity, self-esteem, effective communication and motivation.

### Confirmatory factor analysis 

**Figure 1:**
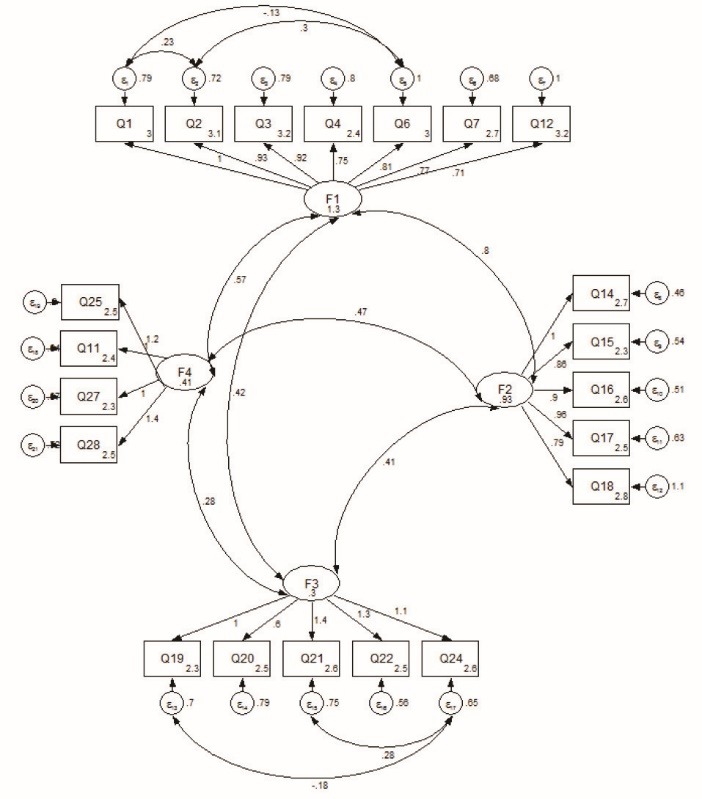
Confirmatory factor analysis (CFA) for student’s emotional growth questionnaire.

Considering the emotional growth as a latent variable with four sub-scales and 21 items, as observed items, we performed CFA (Figure 1).

The modified CFA output illustrated in Figure 1 showed that the solution was appropriate. The following values of absolute fit indices showed an acceptable level of fit: (1) the chi-square test with χ^2^ value of 360.8, *df* = 178, *P* = 0.416, indicated a smaller difference between expected and observed covariance matrices; (2) CFI = 0.914, RMSEA = 0.070 (90% CI: 0.060-0.081) indicated acceptable level of fit.

Table 2 shows the results of internal consistency (Cronbach α) and test–retest reliability (ICC) of four factors and total questionnaire of emotional growth. The results indicated acceptable levels of reliability and repeatability for the questionnaire.

## Discussion

Based on a holistic model of health care, human beings have biological, psychological, social and spiritual dimensions.^20^ Scholars in nursing education should approve latent learning, which occurs simultaneously with the routine educational programs. They should also be able to promote and facilitate the development of various human aspects of the students.^5^ One of the barriers for human growth in nursing students is emotional growth.^21^ Regarding the importance of promoting students’ emotional growth and lack of standard tools for evaluating the emotional growth of students, this study was conducted to devise and provide a standard, valid, and reliable instrument for measuring students’ emotional growth.

In this study, a questionnaire with 21 items was developed. The results of content validity of the questionnaire showed its high relevancy and clarity. EFA was used to assess construct validity using principal component analysis extraction method and Varimax rotation.^22,23^ Based on the EFA, four factors were extracted as follow: positive identity, self-esteem, effective communication and motivation. The factor-structure of the emotional growth questionnaire explained 65.3% of the total variance. Also, the modified CFA showed that the solution was appropriate and in an acceptable level of fit. The intra-class correlation coefficient was used to assess the reliability for numeric items. The ICC 0.61 to 0.8 were considered as good and >0.8 as excellent.^24^ According to the results, all factors approved to be reliable (the ICC value was above 0.64 for the factors).

The study sample size was large enough to conduct a valid EFA.^25,26^ However, the study samples were limited to nursing students; thus, generalizability of the results is limited.

## Conclusion

The developed 21 items questionnaire seems to be a valid and reliable instrument for measuring students’ emotional growth which could be used as a research instrument to evaluate emotional growth educational interventions among university students.

## Ethical approval

The research protocol was approved at the organizational committee of ethics in Baqiyatallh University of Medical Sciences.

## Conflict of interests

The authors declare that there is no conflict of interests.

## Authors’ contributions

All authors contributed to the design of the work and interpretation of the results. The first author conducted the data collection. The first, second and third authors conducted the data analyses. The first and second authors contributed to drafting the manuscript. The third and fourth authors helped in manuscript evaluation. The second and fourth author helped to evaluate and edit the manuscript.

## Supplementary Materials


Supplementary file contains Figure S1.

## Acknowledgments

This manuscript is part of a PhD thesis of Baqiyatallh University of Medical Sciences. The authors would like to greatly acknowledge financial support for this study from Baqiyatallh University of Medical Sciences. They also wish to thank all the participants of this study for their valuable cooperation and participation.

**Table 1:** The Items and 4-factor structure of the student emotional growth questionnaire

**Items**	**Questions**	**Mean**	**SD**	**Factor 1 (positive identity)**	**Factor 2 (self-esteem)**	**Factor 3 (effective communication)**	**Factor 4 (motivation)**
Q1	I feel particularly pleased with the way I am.	4.11	1.33	0.769			
Q2	I feel that life is very rewarding.	3.99	1.24	0.802			
Q3	I wake up feeling rested.	3.81	1.29	0.727			
Q4	I am particularly optimistic about the future.	4.62	1.33	0.693			
Q5	I am committed to ethical principles.	5.09	0.99				
Q6	I think that the world is a good place.	3.99	1.39	0.667			
Q7	There is no gap between what I would like to do and what I have done.	4.23	1.28	0.661			
Q8	I can adapt to different situations.	4.53	1.04				
Q9	I am especially in control of my life.	4.37	1.02				
Q10	I find it easy to make decisions.	3.86	1.20				
Q11	My life is purposeful.	4.35	1.24				0.611
Q12	I have a great deal of energy.	3.99	1.39	0.695			
Q13	I have particularly happy memories of the past.	4.19	1.49				
Q14	I'm as successful as others in my works.	4.35	1.16		0.724		
Q15	There are things in my life that I am proud of.	4.90	1.05		0.629		
Q16	I have enough confidence in my decisions.	4.41	1.08		0.655		
Q17	I certainly feel useless at times.	4.53	1.24		0.631		
Q18	It's easy to talk in the crowd.	4.02	1.46		0.701		
Q19	I love others.	4.49	1.19			0.738	
Q20	I am open to criticism.	4.37	1.10			0.715	
Q21	People love me.	4.45	1.12			0.634	
Q22	I feel good when expressing my feelings, thoughts and ideas to others.	4.49	1.11			0.697	
Q23	People do not abuse me.	4.23	1.23				
Q24	I have a good effect on others.	4.51	1.03			0.633	
Q25	I love what I do.	4.45	1.22				0.569
Q26	I am interested in my field of study.	4.03	1.55				
Q27	I'm trying to reach the goal.	4.74	1.21				0.586
Q28	I welcome a lot of responsibilities.	4.37	1.36				0.687

**Table 2:** Internal consistency and test–retest reliability of four factors and total questionnaire of emotional growth

**Variable**	**Positive identity**	**Self-esteem**	**Effective communication**	**Motivation**	**Total (emotional growth**
Cronbach α	0.88	0.81	0.77	0.78	0.93
ICC (95% CI)	0.91 (0.83 -0.96)	0.75 (0.56 - 0.86)	0.64 (0.40 - 0.79)	0.66 (0.42- 0.81)	0.91 (82.4 -95.6)

## References

[1] Jaeger AJ (2003). Job competencies and the curriculum: an inquiry into emotional intelligence in graduate professional education. Res High Educ.

[2] Harward DW (2007). Engaged learning and the core purposes of liberal education: bringing theory to practice. Liberal Education.

[3] Swaner LE (2007). Linking engaged learning, student mental health and well-being, and civic development: a review of the literature. Liberal Education.

[4] Whitt EJ, Schuh JH, Kinzie J, Kuh GD. Student success in college: Creating conditions that matter. San Francisco, CA: Jossey-Bass; 2013.

[5] Seylani K, Negarandeh R, Mohammadi E (2012). Iranian undergraduate nursing student perceptions of informal learning: a qualitative research. Iran J Nurs Midwifery Res.

[6] Rankin EA, Delashmutt MB (2006). Finding spirituality and nursing presence: the student’s challenge. J Holist Nurs.

[7] Henderson S (2002). Factors impacting on nurses’ transference of theoretical knowledge of holistic care into clinical practice. Nurse Educ Pract.

[8] Tabari Khomeiran R, Deans C (2007). Nursing education in Iran: past, present, and future. Nurse Educ Today.

[9] Nikbakht Nasrabadi A, Emami A, Parsa Yekta Z (2003). Nursing experience in Iran. Int J Nurs Pract.

[10] Christiansen B, Jensen K (2008). Emotional learning within the framework of nursing education. Nurse Educ Pract.

[11] de Araujo Sartorio N, Pavone Zoboli EL (2010). Images of a ‘good nurse’ presented by teaching staff. Nurs Ethics.

[12] Mehrmohammadi M (2004). Curriculum theories.

[13] Hartup WW, RubinZ RubinZ (2013). Relationships and Development.

[14] Duchscher JE (2000). Bending a habit: critical social theory as a framework for humanistic nursing education. Nurse Educ Today.

[15] Perry RN (2009). Role modeling excellence in clinical nursing practice. Nurse Educ Pract.

[16] Nouhi S, Hoseini M, Rokhsarizadeh H, Saburi A, Alishiri G (2012). Progress Motivation among Baqiyatallah University of Medical Sciences students and its relationship with academic achievement. J Mil Med.

[17] Heidari P, Latifnejad R, Sahebi A, Jahaniyan M, Mazloum S (2002). Impact of cognitive behaviour therapy on anxiety level of primary infertile women undergoing IUI. J Reprod Infertil.

[18] Chari MH, Delavarpoor M (2007). Do shy people lack communication skills?. J Iran Psychol.

[19] Liaghatdar MJ, Jafari E, Abedi MR, Samiee F (2008). Reliability and validity of the Oxford Happiness Inventory among university students in Iran. Span J Psychol.

[20] Ehsani SR, Mohamadkhani Ghiasvad A, Mohammadnejad E, Nemati Dopolani F (2015). The concept of spiritual health from the viewpoint of nurses working in intensive care units. J Nurs Midwifery Sci.

[21] Mokhtari Nouri J, Ebadi A, Alhani F, Rejeh N (2014). Growing up and role modeling: a theory in Iranian nursing students’ education. Glob J Health Sci.

[22] Ugulu I (2013). Confirmatory factor analysis for testing validity and reliability of traditional knowledge scale to measure university students’ attitudes. Educ Res Rev.

[23] DeVellis RF (2003). Factor Analysis, Scale Development: Theory and Applications (Applied Social Research Method Series).

[24] Bartko JJ (1966). The intraclass correlation coefficient as a measure of reliability. Psychol Rep.

[25] Williams B, Onsman A, Brown T (2010). Exploratory factor analysis: A five-step guide for novices. Aust J Paramed.

[26] MacCallum RC, Widaman KF, Preacher KJ, Hong S (2001). Sample size in factor analysis: the role of model error. Multivariate Behav Res.

