# Supplementary material for: Development and psychometric evaluation of an emotional growth questionnaire for university students
Source: Health Promot Perspect. 2018 Oct 27;8(4):303–7. doi: 10.15171/hpp.2018.43 (PMC6249497; doi:10.15171/hpp.2018.43)
Supplement: Supplementary file 1 — Supplementary file contains Figure S1. [file hpp-8-303-s001.pdf]

Table S1. Student emotional growth questionnaire

Questions
**Positive identity**
1- I feel particularly pleased with the way I am
2- I feel that life is very rewarding
3- I wake up feeling rested
4- I am particularly optimistic about the future
5- I think that the world is a good place.
6- There is no gap between what I would like to do and what I have done.
7- I have a great deal of energy.
**Self-esteem**
8- I'm as successful as others in my works
9- There are things in my life that I am proud of.
10- I have enough confidence in my decisions.
11- I certainly feel useless at times.
12- It's easy to talk in the crowd.
**Effective communication**
13- I love others.
14- I am open to criticism.
15- People love me.
16- I feel good when expressing my feelings, thoughts and ideas to others.
17- I have a good effect on others.
**Motivation**
18- My life is purposeful
19- I love what I do.
20- I'm trying to reach the goal.
21- I welcome a lot of responsibilities.

Choices and scoring:

"Completely agree"= 6
"Agree"= 5
"Somewhat agree"= 4
"Somewhat disagree"= 3
"Disagree" =2
"Completely disagree" = 1
The potential range of score is21 to 126. The higher score is indicating higher emotional growth.
